# Reconceptualising mindfulness in Cambodia: Khmer translation, cultural adaptation, and validation of the Southampton Mindfulness Questionnaire

**DOI:** 10.3389/fpsyt.2026.1901766

**Published:** 2026-08-19

**Authors:** Alan Maddock, Thearith Heang, Nil Ean, Sisary Kheng, Karen McGuigan, Nerrolyn Ramstrand

**Affiliations:** 1 Department of Health Psychology, Royal College of Surgeons in Ireland, Dublin, Ireland; 2 Exceed Worldwide, Phnom Penh, Cambodia; 3 Department of Prosthetics and Orthotics, National Institute of Social Affairs, Phnom Penh, Cambodia; 4 The Center for Trauma Care and Research Organization (CTRO), Phnom Penh, Cambodia; 5 Department of Psychology, Royal University of Phnom Penh, Phnom Penh, Cambodia; 6 Queen’s Communities and Place, Queens University Belfast, Belfast, United Kingdom; 7 Department of Rehabilitation, Jonkoping University, Jönköping, Sweden

**Keywords:** Cambodia, cross-cultural psychology, cultural adaptation, mindfulness, psychometrics, validation

## Abstract

**Objectives:**

Mindfulness is increasingly used in global mental health, yet its measurement tools are largely developed within Western cultural frameworks. This study aimed to (1) translate and culturally adapt the Southampton Mindfulness Questionnaire (SMQ) into Khmer, (2) evaluate its cultural integrity among Cambodian adults with physical disabilities, and (3) examine the psychometric properties and factor structure of the measure.

**Methods:**

The SMQ was translated using a standardised forward–backward translation process with expert panel review and cognitive interviewing. Exploratory (EFA) and Confirmatory Factor Analyses (CFA) were conducted on responses from 437 Cambodian participants with physical disabilities.

**Results:**

Following cultural adaptation, minor linguistic revisions were made to ensure conceptual equivalence. EFA and CFA supported a 14-item, six-factor structure comprising letting go, acceptance, non-judgement, cognitive-emotional reactivity, absence of aversion, and mindful observation. This structure demonstrated good model fit and internal consistency, diverging from the original Western single-factor model.

**Conclusion:**

This study provides a culturally adapted Khmer version of the SMQ with evidence of structural validity and internal consistency and offers evidence that the structure of mindfulness may vary across cultural contexts. The findings highlight the influence of Buddhist psychological concepts, particularly acceptance and reduced cognitive-emotional reactivity, in shaping the experience of mindfulness in Cambodia. The availability of this scale enables culturally grounded research and clinical practice and supports the development of contextually appropriate mindfulness-based interventions in low- and middle-income settings.

## Introduction

Cambodia has a tragic history of genocide and mass violence. Those who experienced the Pol Pot regime, and others who have lived with its intergenerational consequences, continue to experience high rates of psychological distress, trauma, psychiatric morbidity, and poor physical health (1). This historical legacy, combined with ongoing psychosocial stressors such as multidimensional poverty, contributes to elevated rates of depression, anxiety, and post-traumatic stress disorder (PTSD) within the Cambodian population (2). These risks are further amplified among individuals with physical disabilities, who frequently experience additional barriers to accessing mental health care (1, 3). Indeed, Maddock et al. (1) reported that 35% of Cambodians with physical disabilities were experiencing mild to moderate psychological distress, with 14% experiencing severe distress, and 46% meeting criteria for probable PTSD.

In response to the high burden of mental health difficulties, there is increasing international evidence supporting the effectiveness of mindfulness-based programmes (MBPs) for reducing symptoms of anxiety, depression, and trauma-related distress (4, 5). Mindfulness, originating from Buddhist psychological traditions, is concerned with cultivating present-moment awareness, non-judgement, and acceptance of internal experiences (6). Given that approximately 93% of the Cambodian population identify as Buddhist, with the majority practicing Theravada Buddhism (7), mindfulness-based approaches may be particularly culturally congruent and acceptable in this context. However, despite this apparent alignment, there have been no empirical studies evaluating mindfulness-based interventions in Cambodia (5), limiting the development of evidence-based mental health strategies.

A key barrier to the evaluation and implementation of MBPs in Cambodia is the lack of culturally appropriate and psychometrically valid measurement tools (1). While numerous mindfulness questionnaires have been developed in English-speaking contexts, few have been translated and culturally adapted using rigorous methods that ensure equivalence between source and target versions (1, 8). Importantly, the process of translation extends beyond linguistic conversion and must account for semantic, idiomatic, experiential, and conceptual equivalence to ensure that items retain their intended meaning across cultural contexts (9, 10). Without such adaptation, there is a risk that psychological constructs may be misinterpreted, thereby limiting the validity and utility of measurement tools in non-Western populations.

In addition to linguistic challenges, there is growing recognition within cultural psychiatry that psychological constructs cannot be assumed to have universal meanings across contexts. Kleinman (11) described this as a “category fallacy, “ whereby constructs identified across cultures are assumed to represent the same underlying phenomena, even when their meanings differ. Similarly, Kirmayer (12) and Kirmayer and Minas (13) have argued that psychiatric knowledge and practice are themselves shaped by cultural systems of meaning, and that symptoms, experiences, and coping processes are embedded within specific social and cultural contexts. Cultural psychiatry research consistently demonstrates that mental health phenomena—including distress, cognition, and emotion regulation—are influenced by culturally mediated interpretations, social practices, and local explanatory models (14).

Mindfulness provides a particularly important example of this issue. Although it is rooted in Buddhist traditions, contemporary psychological conceptualisations and measurements of mindfulness have largely been developed within Western scientific frameworks (15). As a result, existing measures may prioritise certain aspects of mindfulness (e.g., attention and awareness), while underrepresenting others that are central within Buddhist psychology, such as acceptance, non-attachment, and insight into the transient nature of experience (16). This raises the possibility that the structure and meaning of mindfulness may differ when assessed in cultural contexts such as Cambodia, where Buddhist philosophical traditions shape everyday understandings of cognition, emotion, and selfhood.

Several self-report measures have been developed to assess mindfulness, including the Five Facet Mindfulness Questionnaire (FFMQ), the Mindful Attention Awareness Scale (MAAS), and the Cognitive and Affective Mindfulness Scale-Revised (CAMS-R). However, these measures were designed to assess broad trait mindfulness and place considerable emphasis on attention, awareness, and general mindfulness tendencies (17). In contrast, the Southampton Mindfulness Questionnaire (SMQ) was specifically developed to assess how individuals respond to distressing thoughts and images, focusing on processes such as decentering, non-reactivity, and non-judgement in the context of psychological distress (18). Given the high prevalence of trauma exposure, psychological distress, and PTSD within Cambodia, particularly among people with physical disabilities (1, 2), the SMQ was considered especially relevant for the present study. Its focus on individuals’ relationships with distressing cognitions makes it well suited to examining mindfulness processes within populations affected by trauma and adversity.

The Southampton Mindfulness Questionnaire (SMQ) (18) is a 16-item self-report measure designed to assess how individuals relate to distressing thoughts and images. It focuses on key psychological processes such as non-reactivity, non-judgement, and decentered awareness, which are central to cognitive models of mental health (18). The SMQ has demonstrated good reliability and validity in previous research, including strong internal consistency and significant correlations with other measures of mindfulness (17, 18). Its emphasis on individuals’ responses to distressing cognitions makes it particularly suited for clinical populations, including those experiencing anxiety, depression, and trauma-related disorders.

Despite its potential utility, the SMQ has not been translated or validated for use in Cambodia or other Khmer-speaking populations. Furthermore, existing evidence regarding its factor structure has been mixed, and there is a need for further validation across diverse cultural contexts (19). Given the cultural and contextual differences that may shape the experience and expression of mindfulness, it is important to examine whether the SMQ retains its psychometric properties when applied in Cambodia, or whether alternative factor structures emerge.

This study had two primary aims: (1) to use a standardised method to translate and culturally adapt the Southampton Mindfulness Questionnaire (SMQ) into Khmer, (2) to evaluate its cultural integrity among Cambodian adults with physical disabilities, and (3) to examine the psychometric properties and factor structure of the translated SMQ using exploratory and confirmatory factor analyses.

## Methods

### Study design

This study employed a cross-sectional design to translate, culturally adapt, and validate a Khmer version of the Southampton Mindfulness Questionnaire (SMQ). The study combined established procedures for cross-cultural adaptation with psychometric evaluation using exploratory and confirmatory factor analyses.

### Ethics statement

This study was conducted in accordance with the principles of the Declaration of Helsinki. Ethical approval was obtained from the National Ethics Committee for Health Research in Cambodia (Approval number: #207 NECHR). All participants provided informed consent prior to participation. Study procedures were designed to ensure accessibility for individuals with physical disabilities and varying levels of literacy, including the option to complete the questionnaire with assistance where required. Participant confidentiality and anonymity were maintained throughout the study, and all data were handled in accordance with ethical guidelines.

### Translation and cultural adaptation

**Figure 1:**
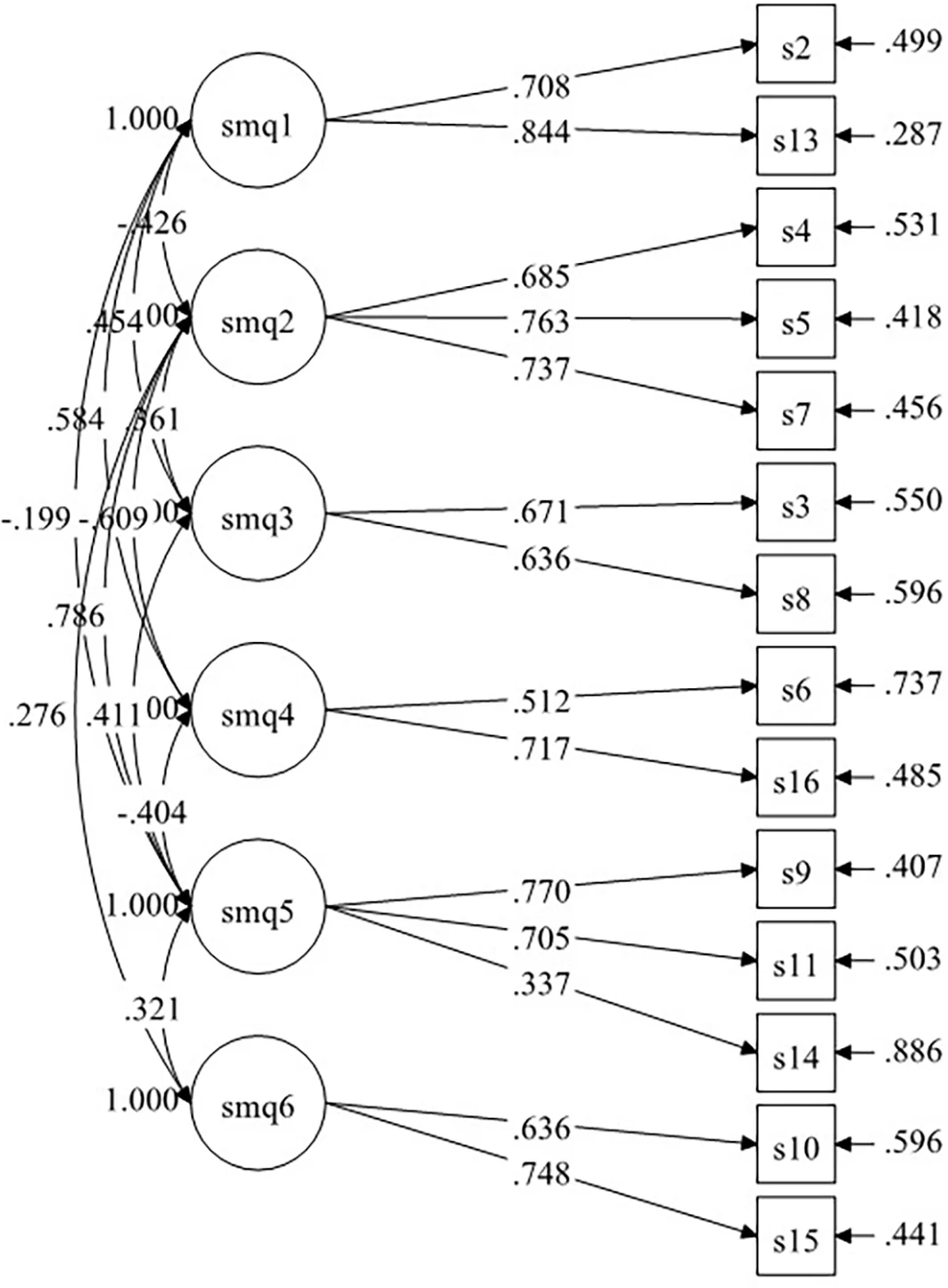
Six factor model (Model 3b) based on data from the Khmer translated SMQ.

The translation and cultural adaptation of the SMQ followed the Functional Assessment of Chronic Illness Therapy (FACIT) translation and linguistic validation method (20), in addition to the World Health Organization guidelines on the translation of instruments (21). The process, illustrated in **Figure 1** (see **Supplementary File 1**), involved three phases: (1) reconciliation of two independent translations, (2) evaluation of the reconciled document against the original version, and (3) pre-testing of the translation in the target context. The overall aim of this process was to ensure semantic, idiomatic, experiential, and conceptual equivalence between the original English version and the Khmer translation (9, 10).

#### Phase 1: forward translation and reconciliation

Two independent Khmer translations of the SMQ were generated by native Khmer speakers. Translation A was completed by a Cambodian clinical psychologist, while Translation B was conducted by a group of four allied health professionals who provide clinical care to people with disabilities.

Prior to translation, all translators were provided with verbal and written guidance based on World Health Organization (WHO) guidelines (21). These instructions emphasised the importance of producing a clear and accessible translation that prioritised conceptual equivalence rather than a word-for-word rendering of the original English version.

A reconciliation workshop was subsequently held to synthesise both translations into a single version. Participants in this workshop included 15 Cambodian allied health professionals (prosthetist/orthotists), one Cambodian clinical psychologist, and two native English-speaking professionals (an allied health professional and a social worker who is also a Lecturer in Psychology with expertise in mindfulness interventions). The reconciliation process followed established decision criteria (22).

#### Phase 2: back translation and expert review

The reconciled Khmer version of the SMQ was back-translated into English by a professional medical translator who is a native English speaker and was not involved in the initial translation process and was blinded to the original instrument. The back-translated version was reviewed by the research team (three native Khmer speakers and two native English speakers) to identify discrepancies, ambiguities, or inaccuracies. Where issues were identified, the team engaged in iterative discussion to refine the Khmer version. Particular attention was given to terms that did not have direct linguistic equivalents in Khmer, ensuring that conceptual meaning was preserved while maintaining clarity for respondents.

#### Phase 3: cognitive interviewing

Cultural validity of the Khmer SMQ was assessed using cognitive interviewing techniques (23). Participants were recruited from three prosthetic and orthotic clinics in Cambodia. Inclusion criteria required participants to be aged 18 years or older and to have a physical disability.

Purposive sampling was used to ensure representation of participants from both urban and rural areas, and across a range of literacy levels. Two rounds of cognitive interviews were conducted by five Cambodian allied health professionals who received training in cognitive interviewing prior to data collection.

A written manual was provided to each interviewer, including standardised instructions and scripted probes to guide data collection. During each interview, participants completed the Khmer SMQ independently or, where necessary, had items read aloud by the interviewer. After responding to each item, participants were asked to describe how they interpreted the question and how they arrived at their response. Probing questions focused on comprehension, memory retrieval, decision-making processes, and response selection.

Interviewers completed a structured feedback sheet following each interview, recording participant characteristics (e.g., age, gender) and documenting any issues identified with specific SMQ items. After the first round of interviews, interviewers met to review the feedback and identify problematic items. Necessary revisions were made to the questionnaire, and these modifications were tested in a second round of interviews. No further issues requiring modification were identified following the second round.

### Participants and data collection

A total of 437 participants were recruited from prosthetic and orthotic clinics in Cambodia between March 2023 and February 2024. Participants completed the Khmer version of the SMQ using a digital survey platform (Qualtrics). Where required, trained health professionals assisted participants in completing the questionnaire.

The sample included individuals from both urban and rural areas and represented a wide age range. Of the total sample, 358 participants were male and 79 were female. The largest age group was 55–64 years (n = 124), while the smallest was 18–24 years (n = 26). Additional age groups included 25–34 years (n = 44), 35–44 years (n = 103), 45–54 years (n = 81), and 65 years or older (n = 59). The predominance of male participants reflects the demographic profile of individuals accessing prosthetic and orthotic services in Cambodia. Within the sample, 69 reported they were single (15.8%), 340 married (78.0%), 15 divorced and 12 widowed (3.4% and 2.8% respectively). Of the 428 participants reflecting their location, 175 lived in rural areas (40.9%), with the remaining 253 living in urban locations (59.1%). Education level varied among respondents, with 77 reporting they had no education (17.7%), 139 with primary education (32.0%), 108 secondary level (24.8%), 60 completing high school (13.8%) and 51 attending college or further education (11.7%). Employment status was reflected as Housewife (n=28, 6.5%), 234 were business owners (53.9%), 104 were employees (24.0%), 10 were students (2.3%), with 58 reporting they were unemployed (13.4%). Living situation was also reported, with 25 living alone (5.8%), 398 living with family (92.1%) 5 living with friends (1.2%) and 4 reporting other living arrangements (0.9%). The cause of their disability varied greatly across the sample, with landmine explosions, accidents, and acquired deformities the most commonly reported causes (n=195, 44.7%; n=111, 26.5%; n=61, 14.0% respectively).

### Data analysis

Data were analysed using IBM SPSS Statistics (Version 29), with structural equation modelling conducted using Mplus Version 8.5 (24).

In line with best practice recommendations, the sample was divided into two independent datasets based on the date of questionnaire completion (25). Exploratory Factor Analysis (EFA) was conducted using the first dataset (n = 218), while Confirmatory Factor Analysis (CFA) was conducted using the second dataset (n = 219).

### Exploratory factor analysis

EFA was conducted using maximum likelihood estimation (ML) with GEOMIN rotation (24). ML assumes independent observations, an appropriately specified model, and adequate sample size. The SMQ items were treated as continuous because they used a likert response scale. Missing data (<1%) were handled using full-information maximum likelihood, which uses all available data under the assumption that missingness is ignorable or missing at random conditional on variables included in the model. Multiple factor solutions were examined to identify the underlying structure of the Khmer SMQ. Model selection was guided by factor loadings, communalities, and interpretability. Items with factor loadings below 0.32 were considered for removal in line with established guidelines (26).

### Confirmatory factor analysis

CFA was conducted using structural equation modelling to evaluate three competing models:

- Model 1: Single-factor model
- Model 2: Four-factor model based on the original SMQ structure, specifying the 4 existing subscales of Mindful observation (SMQ-MO); Non-attachment (SMQ-NA); Aversion (SMQ-Av); and Non-judgement (SMQ-NJ)
- Model 3: Factor structure identified through EFA

Model parameters were estimated using full information maximum likelihood based on covariance matrices.

### Model fit assessment

Model fit was assessed using multiple indices, including:

- Chi-square (χ²)
- Comparative Fit Index (CFI)
- Tucker–Lewis Index (TLI)
- Root Mean Square Error of Approximation (RMSEA)
- Standardised Root Mean Square Residual (SRMR)

Acceptable model fit was was assessed using these indices in line with guidelines set out by Hoyle and Panter (27); with a non-significant chi-square, CFI and TLI ≥ 0.90, RMSEA ≤ 0.05, and SRMR ≤ 0.08 (27–31). Comparative model fit was evaluated using Akaike Information Criterion (AIC) (32) and Bayesian Information Criterion (BIC) (33). The BIC (33) is recognised as a good indicator of comparative model fit (34, 35) with lower values indicating better fit.

### Reliability analysis

Internal consistency of the scale and its subscales was assessed using Cronbach’s alpha coefficients. Values of 0.70 or above were considered indicative of acceptable reliability. In line with previous recommendations, lower values (≥ 0.60) were considered acceptable for subscales comprising a small number of items in an exploratory research context (36).

## Results

### Translation and cultural adaptation

Two independent Khmer translations of the SMQ were reviewed during a reconciliation workshop. For 12 of the 16 items, the wording from Translation B was retained, as it was judged to better reflect the conceptual meaning of the original English items. The remaining four items were identical across both translations and were retained without modification.

Back-translation identified issues in seven items, primarily relating to conceptual equivalence. Revisions were made to four items following expert panel discussion (see **Supplementary File 2**). A recurring issue concerned the translation of the term “judge, “ which was interpreted in Khmer as having legal connotations rather than meaning evaluative or reflective judgement. Similarly, the term “unpleasant” was initially translated as “uncomfortable, “ which did not fully capture the intended meaning. These terms were subsequently revised to better reflect their conceptual intent.

During the first round of cognitive interviews (n = 10), participants demonstrated difficulty understanding item 8 “*I judge myself as good or bad, depending on what the thought/image is about*”, particularly in distinguishing between evaluating a thought and evaluating the self more generally. This item was rephrased to emphasise that responses should relate to specific thoughts or images. In addition, the introductory statement (“Usually when I experience distressing thoughts and images…”) was repeated before each item, as participants found the original format difficult to follow.

A second round of cognitive interviews (n = 6) confirmed that all items were comprehensible and culturally appropriate, with no further issues identified. The final Khmer version of the SMQ was deemed linguistically and culturally valid for use in the target population. The final version of the SMQ in Khmer is contained in **Supplementary File 3**.

### Descriptive statistics

Descriptive statistics for the 16 SMQ items are presented in **Table 1**. Mean scores were generally moderate to high across items, with negative skewness observed for the majority of items, indicating a tendency toward higher endorsement of mindfulness-related responses. Absolute skewness values below 2 and absolute kurtosis values below 7 were treated as indicating no severe univariate non-normality for maximum-likelihood structural equation modelling (37, 38). The observed skewness values ranged from −1.614 to.223, and kurtosis ranged from −1.333 to 3.762, so falling within the specified range.

**Table 1:** Descriptive statistics based on the Khmer sample (n=437).

Item	Mean	Variance	Skewness	Kurtosis
S1	4.622	2.359	-.778	-.748
S2	4.119	2.393	-.303	-1.333
S3	4.961	1.282	-1.323	1.333
S4	5.211	1.155	-1.510	2.187
S5	5.119	1.157	-1.351	1.576
S6	4.195	2.079	-.251	-1.137
S7	5.082	1.014	-1.335	1.997
S8	4.822	1.465	-1.189	.526
S9	5.278	1.118	-1.490	2.208
S10	4.799	1.758	-1.047	.085
S11	5.348	0.781	-1.614	3.762
S12	4.442	1.624	-.702	-.584
S13	4.259	2.160	-.508	-1.027
S14	5.232	1.109	-1.438	2.229
S15	4.723	1.477	-.973	.287
S16	3.592	2.310	.223	-1.291

### Exploratory factor analysis

Exploratory factor analysis (EFA) was conducted on the first dataset (n = 218). Communalities, following extraction, were assessed to ensure appropriate sample size for analysis, with all reported above 0.5, thus the sample size is sufficient for analysis (39). The Kaiser-Meyer-Olkin (KMO) measure verified sampling adequacy as marvellous or superb (40): KMO = .985, with Bartlett’s Test of Sphericity found to be significant, χ²(120) = 1758.926, p <.001, reflecting the data set was suitable for factor analysis. Initial analyses indicated that one- to four-factor solutions did not provide an adequate fit to the data (see **Table 2**). Progressive extraction of additional factors resulted in improved model fit, with the six-factor solution demonstrating the best overall fit (χ² = 46.683; df = 39; p = 0.186; CFI = 0.996; TLI = 0.988; RMSEA = 0.021; SRMR = 0.015). As shown in **Table 2**, model fit improved consistently with the addition of factors, with the six-factor model yielding the lowest AIC and BIC values, as well as superior fit indices across all metrics.

**Table 2:** EFA Model fit statistics.

Model (no. of factors)	*χ^2^*	CFI	TLI	RMSEA	SRMR	AIC	BIC
Model 1 (1)	1067.883	.494	.416	.146	0.128	21769.276	21965.113
Model 2 (2)	557.132	.754	.669	.110	0.077	21288.525	21545.561
Model 3 (3)	262.809	.901	.842	.076	.040	21022.202	21336.357
Model 4 (4)	157.421	.950	.903	.059	.029	20942.815	21310.009
Model 5 (5)	86.469	.981	.954	.041	.020	20895.862	21312.016
Model 6 (6)	46.683	.996	.988	.021	.015	20878.076	21309.109

The six-factor solution was the only model to produce a non-significant chi-square value and also demonstrated the lowest Akaike Information Criterion (AIC) and Bayesian Information Criterion (BIC), indicating superior model fit relative to alternative solutions.

**Figure 2:**
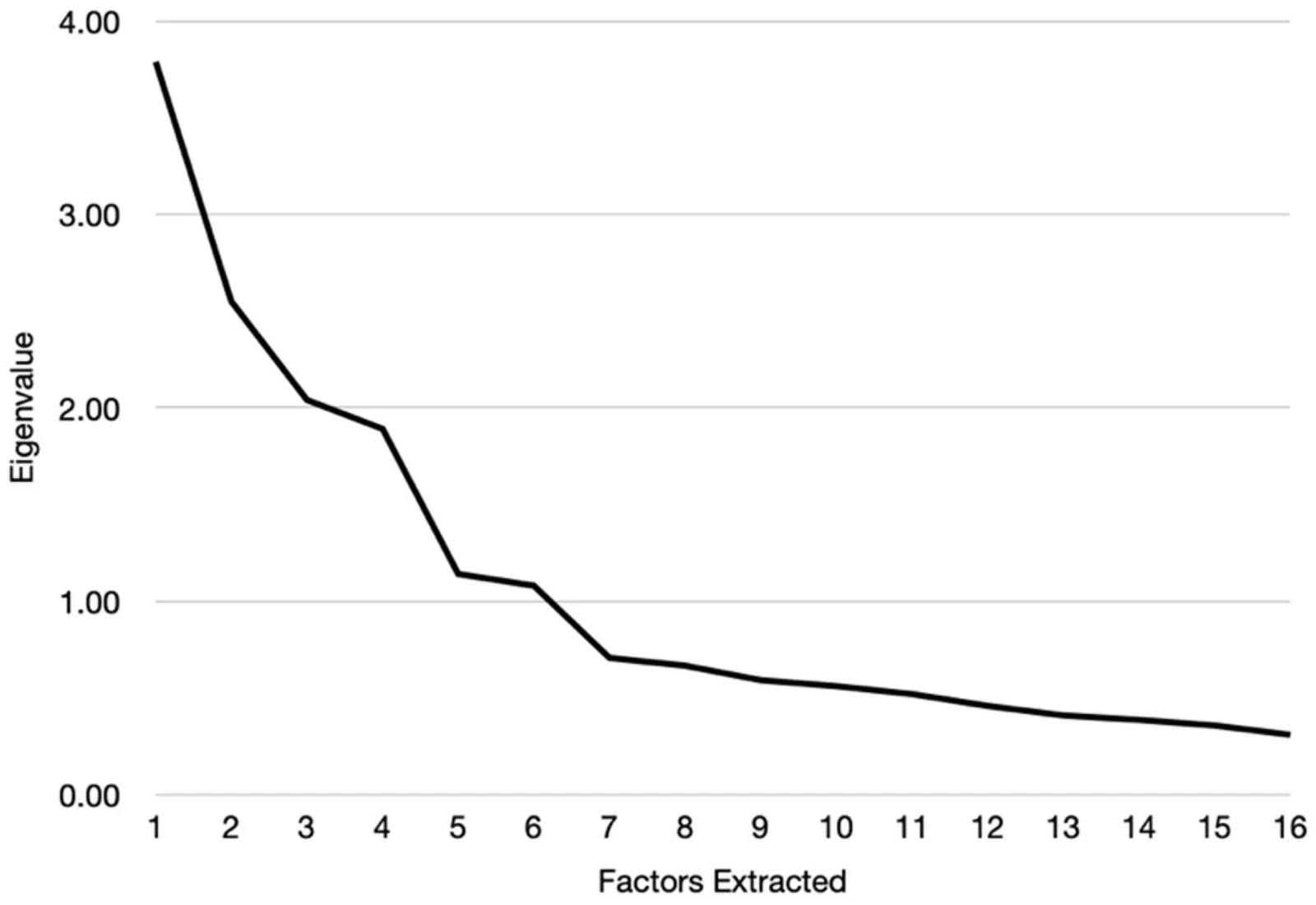
Scree plot.

This corresponds to the traditional Kaiser-Guttman rule for retaining eignevalues greater than 1 (41–43), which infers the inclusion of 6 factors (eigenvalues ranging from 3.794 to 1.089). Further interpretation of the accompanying scree plot, reflects an ‘elbow’ highlighting the inclusion of six factors (see **Figure 2**).

Lastly, a Monte Carlo parallel analysis was conducted (44), which confirmed the proposed included six factors had eigenvalues greater than the corresponding simulated eigenvalues at the 95^th^ percentile (simulated eigenvalues ranging from 1.340 to 1.084). As such, six factors were retained, accounting for 63.539% of the variance (See **Table 3**).

**Table 3:** Variance explained by the 6 factor model.

Factor/component	% of variance explained	Cumulative variance explained
Factor 1	19.350	19.350
Factor 2	14.848	34.198
Factor 3	9.748	43.946
Factor 4	7.335	51.281
Factor 5	6.304	57.585
Factor 6	5.953	63.539

The factor loadings for the six-factor model are presented in **Table 4**. To ensure a parsimonious and interpretable solution, items with factor loadings below 0.32 were removed in line with best practice guidance (26). The resulting factor structure comprised six interpretable factors, reflecting distinct dimensions of mindfulness within the sample.

**Table 4:** Estimated factor loadings and communalities after extraction.

Item	Factor 1 (SMQ1)	Factor 2 (SMQ2)	Factor 3 (SMQ3)	Factor 4 (SMQ4)	Factor 5 (SMQ5)	Factor 6 (SMQ6)
S1						.482 (.372)
S2	.594 (.622)					
S3			.541 (.610)			
S4		.717 (.600)				
S5		.535 (.664)				
S6				.931 (.527)		
S7		.351 (.623)				
S8			.781 (.580)			
S9					.677 (.690)	
S10						.996 (.697)
S11					.788 (.519)	
S12			.398 (.187)			
S13	.458 (.650)					
S14					.322 (.687)	
S15						.390 (.684)
S16				.325 (.503)		

**Table 4** also highlights high communality the majority of the are acceptable (≥ 0.40) or high (> 0.60) suggesting these explain the factor well, and should be retained (39, 45). However, items 1 and 12 have less than acceptable, or low, communality which suggests they may need to be removed from future analyses (39, 46).

### Confirmatory factor analysis

Confirmatory factor analysis (CFA) was conducted on the second dataset (n = 219) to evaluate three competing models:

1. a single-factor model,
2. a four-factor model based on the original SMQ structure, and
3. a six-factor model derived from the EFA findings.

Initial testing of the six-factor model including all 16 items (Model 3a) indicated suboptimal fit relative to a revised model. In the EFA, items 1 and 12 had primary loadings of.482 and.398, respectively; with low communality reported for both, suggesting need to remove these. In the independent CFA sample, neither item loaded significantly on its specified factor. Their removal produced a marked improvement in global and local model fit (see **Table 5**). As such, these items were removed, resulting in a revised six-factor model (Model 3b). No further model modifications were introduced during CFA, nor were modifications suggested by MPlus.

**Table 5:** CFA Model fit statistics.

Model (no. of factors)	*χ^2^*	CFI	TLI	RMSEA (90%CI)	SRMR	AIC	BIC
Model 1 (1)	1054.958	.496	.419	.145 (.137-.153)	.127	21584.144	21779.540
Model 2 (4)	991.275	.527	.421	.145 (.137-.154)	.138	21532.462	21752.282
Model 3a (6) 16 items	324.433	.875	.832	.078 (.069-.087)	.074	20883.620	21140.076
Model 3b (6) 14 items	150.602	.944	.918	.057 (.046-.069)	.047	18044.106	18276.269

Model fit statistics for all models are presented in **Table 5**. The final six-factor, 14-item model (Model 3b) demonstrated acceptable fit to the data (χ² = 150.602; CFI = 0.944; TLI = 0.918; RMSEA = 0.057; SRMR = 0.047) and provided the best overall fit among the models tested, as indicated by the lowest AIC and BIC values. All retained factor loadings were statistically significant (p <.05).

### Factor structure interpretation

All latent factors were significantly correlated (range = 0.121–0.786, p <.05), supporting the conceptualisation of mindfulness as a multidimensional construct.

The six-factor structure identified in the Khmer SMQ is presented in **Table 6**. The factors corresponded to conceptually distinct dimensions of mindfulness:

**Table 6:** Standardised factor loadings and standard errors obtained in CFA.

Factor	Estimate	*SE*	*P*-value
Factor 1: Letting go			
They take over my mind for quite a while afterwards	.708	.037	.000
I keep thinking about the thought or image after it’s gone	.844	.036	.000
Factor 2: Acceptance			
I feel calm soon after	.685	.032	.000
I am able to accept the experience	.763	.027	.000
I notice how brief the thoughts and images really are	.737	.029	.000
Factor 3: Non-Judgement			
I judge the thought/image as good or bad	.671	.045	.000
I judge myself as good or bad, depending on what the thought/image is about	.636	.045	.000
Factor 4: Cognitive-emotional reactivity			
I get angry that this happens to me	.512	.052	.000
I lose myself in the thoughts/image	.717	.058	.000
Factor 5: Absence of Aversion			
I ‘step back’ and am aware of the thought or image without getting taken over by it	.770	.033	.000
I accept myself the same whatever the thought/image is about	.705	.034	.000
I find it so unpleasant I have to distract myself and not notice them	.377	.049	.000
Factor 6: Mindful observation			
I just notice them and let them go	.636	.061	.000
I try just to experience the thoughts or images without judging them	.748	.067	.000

- Factor 1: Letting go (attachment to thoughts and images)
- Factor 2: Acceptance
- Factor 3: Non-judgement
- Factor 4: Cognitive and emotional reactivity
- Factor 5: Absence of aversion
- Factor 6: Mindful observation

Four of these factors corresponded broadly to the constructs underpinning the original SMQ (18), while two additional factors—acceptance and cognitive-emotional reactivity—emerged as distinct dimensions in this sample. Rather than representing a purely statistical subdivision of the original SMQ, the six-factor structure is theoretically consistent with both contemporary mindfulness theory and Buddhist psychological models. Mindfulness has increasingly been conceptualised as a multidimensional construct comprising related but distinct processes, including non-judgement, decentring, acceptance, non-attachment, and emotion regulation (15, 47). Within Buddhist psychology, acceptance and reduced reactivity are not viewed as secondary consequences of mindfulness but as core mechanisms through which suffering is reduced (6). The emergence of these dimensions as distinct factors therefore suggests that Cambodian respondents may differentiate between observing experiences, accepting them, and regulating emotional responses, reflecting a more nuanced organisation of mindfulness than that captured by the original unidimensional model.

Internal consistency of the 14-item Khmer SMQ was acceptable (Cronbach’s α = 0.72). Cronbach’s alpha values for the six multi-item factors ranged from 0.6 to 0.76. While some factors demonstrated good internal consistency, acceptable reliability observed in others likely reflects the limited number of items per factor and the complexity of the construct being assessed. For a more robust measure of internal consistency, McDonald’s Omega was calculated for the 14-item scale (In the present sample, internal consistency was good (ω*_t_* = 0.799). However, Composite Reliability (CR) and Average Variance Extracted (AVE) suggest mixed convergent validity across the six factors (See **Table 7**).

**Table 7:** Composite reliability (CR) and average variance extracted (AVE).

Factor/component	CR	AVE
Letting Go	0.75	0.61
Acceptance	0.77	0.53
Non-Judgement	0.60	0.43
Cognitive-Emotional Reactivity	0.55	0.39
Absence of Aversion	0.67	0.41
Mindful Observation	0.65	0.48

CR exceeded the recommended threshold of 0.70 for Letting go (CR = 0.75) and Acceptance (CR = 0.77), while Absence of aversion (CR = 0.67), Mindful observation (CR = 0.65), and Non-judgement (CR = 0.60) demonstrated moderate reliability. A lower estimate was observed Cognitive-emotional reactivity (CR = 0.55). AVE exceeded the recommended criterion of.50 for Letting go (AVE = 0.61) and Acceptance (AVE = 0.53), indicating adequate convergent validity for these factors. However, Fornell and Larcker (48) suggested that AVE values below 0.50 may be considered acceptable when composite reliability exceeds 0.60. On this basis, Absence of aversion, Mindful observation, and Non-judgement factors may be considered provisionally acceptable.

Additionally discriminant validity was evaluated using the Fornell-Larcker criterion (48), with five of the six constructs satisfying this i.e. √AVE exceeds it’s highest latent/absolute correlation. Letting Go, Acceptance, Non-Judgement, and Mindful Observation, indicated clear discriminant validity; with Cognitive-emotional reactivity marginal as it only slightly exceeded its strongest correlation. Absence of Aversion failed to satisfy the Fornell–Larcker criterion, as its strongest correlation with Acceptance (r=0.69) exceeded its √AVE (.64), suggesting some possible overlap between these two constructs.

Overall, the findings support a culturally adapted 14-item, six-factor structure of the Khmer SMQ, demonstrating good model fit and internal consistency. This structure differs from the original English version, suggesting cultural variation in the organisation of mindfulness. Taken together, these findings indicate that mindfulness is best represented as a multidimensional construct in this Cambodian sample.

## Discussion

This study aimed to translate and culturally adapt a Khmer version of the Southampton Mindfulness Questionnaire (SMQ), evaluate its cultural integrity among Cambodian adults with physical disabilities, and examine its psychometric properties and factor structure. The findings provide evidence of structural validity and internal consistency for the Khmer SMQ, while suggesting that the organisation of mindfulness may differ from that identified in previous Western studies.

Specifically, exploratory and confirmatory factor analyses supported a 14-item, six-factor model, rather than the single-factor structure originally proposed by Chadwick et al. (18).

These findings contribute to a growing body of literature suggesting that psychological constructs are not universally invariant, but are shaped by cultural systems of meaning (15, 19). The present findings are consistent with the concept of “category fallacy” (11), demonstrating that mindfulness, when examined in a Cambodian context, is organised in a way that reflects locally meaningful distinctions between cognitive and emotional processes. The identification of six distinct but related factors—including letting go, acceptance, non-judgement, cognitive-emotional reactivity, absence of aversion, and mindful observation—suggests that mindfulness is best conceptualised as a multidimensional construct in this context. This finding is theoretically consistent with contemporary mindfulness models, which conceptualise mindfulness as comprising several related but distinct processes, including attention, decentring, non-judgement, acceptance, non-attachment, and emotion regulation, rather than a single underlying dimension (15, 47). While four of these factors broadly align with the constructs underpinning the original SMQ (18), two additional dimensions—acceptance and cognitive-emotional reactivity—emerged as distinct factors in the Khmer version. This divergence is consistent with previous critiques of unidimensional models of mindfulness, which argue that mindfulness is inherently multifaceted and contextually shaped (15, 19). The present findings also differ from previous validation studies of the SMQ. Chadwick et al. (18) originally conceptualised the SMQ as a unidimensional measure of mindful responding to distressing thoughts and images, whereas the Khmer version demonstrated a six-factor structure. Similarly, the German validation reported by Böge et al. (49) largely supported the original conceptualisation of the instrument and did not identify the emergence of distinct acceptance or cognitive-emotional reactivity factors. However, the German validation was conducted in a different cultural and clinical context, making direct comparisons necessarily tentative. The present findings therefore suggest that the organisation of mindfulness-related processes may vary across contexts and populations. However, differences between studies may also reflect variations in sample characteristics, translation processes, clinical status, and methodological approaches to factor extraction. Further cross-cultural validation studies are required to determine whether the multidimensional structure identified in the Khmer version represents a culturally specific pattern or a broader feature of mindfulness measurement that has not been fully captured by previous models.

The emergence of acceptance as a distinct factor is particularly noteworthy. Acceptance is widely regarded as a central component of Buddhist psychological traditions (6), where it is understood as a non-judgemental openness to experience and an awareness of the transient nature of thoughts and emotions. Although present within many Western conceptualisations of mindfulness, it is not explicitly represented as a separate dimension in the original SMQ. Its prominence in the Khmer version suggests that Cambodian participants may draw more strongly on culturally embedded, Buddhist-informed understandings of cognition and emotional regulation when responding to items. This finding aligns with broader theoretical work highlighting the centrality of acceptance and non-attachment in contemplative traditions (47). While the emergence of acceptance as a distinct factor may reflect the influence of Buddhist psychological concepts within Cambodian culture, it is also possible that this finding was influenced by characteristics of the study sample, including high levels of trauma exposure, psychological distress, and the experience of living with a physical disability.

The identification of cognitive and emotional reactivity as a distinct factor highlights a culturally meaningful dimension of how individuals relate to distressing internal experiences. Reduced reactivity is a key mechanism within both mindfulness-based interventions and Buddhist contemplative practices, where it is associated with reduced attachment and improved emotional regulation (50). The emergence of this construct as a standalone factor suggests that it may represent an independently salient component of mindfulness in this population. Similarly, the prominence of cognitive-emotional reactivity may reflect both culturally informed understandings of emotional regulation and the specific experiences of a clinical population exposed to adversity and ongoing psychosocial stressors.

Taken together, these findings can be understood within broader cultural psychiatry frameworks, which emphasise that experiences of distress, cognition, and coping are embedded within cultural and social contexts. Kirmayer and Minas (13) argue that both the expression of mental health difficulties and the processes through which individuals regulate emotion are shaped by culturally mediated interpretations and practices. More recent perspectives further highlight that psychological processes arise through dynamic interactions between individuals, social environments, and systems of cultural knowledge (12, 14). From this perspective, the multidimensional structure identified in the Khmer SMQ may reflect culturally grounded ways of understanding and responding to internal experiences, shaped by both Buddhist philosophy and the lived realities of individuals in Cambodia.

Patient-reported outcome measures are recognised as important sources of information for both clinical research and routine clinical care. However, their availability and use in low- and middle-income countries (LMICs) remains limited (51). This lack of culturally validated measures restricts the ability to evaluate interventions, examine mechanisms of change, and include diverse populations in multinational research (52). The present study addresses this gap by providing a psychometrically robust Khmer measure of mindfulness, supporting both research and clinical practice in Cambodia and contributing to efforts to reduce global mental health inequities in line with Sustainable Development Goal 3.

These findings have important implications for clinical practice and intervention development. Mindfulness-based programmes (MBPs) have demonstrated effectiveness in reducing symptoms of anxiety, depression, and post-traumatic stress across a range of populations (4, 5). However, the theoretical frameworks underpinning MBPs are largely derived from Western models of cognition and may not fully capture culturally specific processes. The present findings suggest that, in the Cambodian context, therapeutic mechanisms such as acceptance and reduced cognitive-emotional reactivity may be particularly central. As such, culturally adapted MBPs that explicitly emphasise these processes may enhance engagement and effectiveness.

A key strength of this study was the use of a systematic, multi-stage translation and cultural validation process, incorporating forward–backward translation and cognitive interviewing (52). The inclusion of local stakeholders and attention to literacy ensured that the measure was accessible and meaningful within the Cambodian context. This is particularly important given the complexity of the mindfulness construct and the need to maintain conceptual equivalence across cultural settings (9).

Despite these strengths, several limitations should be acknowledged. First, although a rigorous and systematic approach to translation and cultural validation was employed, cognitive interviews were conducted by allied health professionals with limited experience in this methodology. While structured guidance was provided (23), it is possible that some nuances in participant understanding were not fully captured. Second, the sample was drawn exclusively from individuals receiving prosthetic and orthotic services, which limits the generalisability of the findings to the broader Cambodian population. Individuals with physical disabilities may experience unique psychosocial challenges, including higher rates of trauma exposure, psychological distress, social exclusion, and healthcare needs, which could influence how mindfulness is understood and reported. Consequently, the factor structure identified in the present study may not fully reflect the organisation of mindfulness among other Cambodian populations. The sample was also characterised by a substantial gender imbalance, with males comprising more than 80% of participants. While this reflects the demographic profile of individuals accessing prosthetic and orthotic services in Cambodia, it may have influenced the observed factor structure. Previous research suggests that mindfulness-related constructs and emotional processing may vary across demographic groups. Consequently, the extent to which the present findings generalise to Cambodian women remains unclear. Future studies should seek to recruit more gender-balanced samples and examine whether the factor structure of the Khmer SMQ is consistent across gender groups. Indeed, more generally, measurement invariance was not examined in this current study. The gender imbalance and limited number of participants available within age and residence subgroups precluded adequately powered analyses in this instance. Consequently, it cannot yet be assumed that the Khmer SMQ operates equivalently across gender, age, location or disability groups. Future studies should seek to recruit larger, more balanced samples to address these aspects. Third, while the study established structural validity, internal consistency, convergent and discriminant validity; additional psychometric properties—including test–retest reliability, predictive validity, responsiveness, and measurement invariance were not assessed and should be examined in future research. In addition, several factors demonstrated only modest internal consistency. Evidence supporting convergent validity was mixed across the model. Letting go and Acceptance demonstrated satisfactory CR and AVE. Although AVE values for Absence of aversion, Mindful observation, and Non-judgement were below the conventional.50 criterion, their composite reliability estimates exceeded.60, consistent with the guidance of Fornell and Larcker (48), suggesting that these constructs demonstrate acceptable convergent validity for an initial validation study. The relatively weaker performance of Cognitive-emotional reactivity is likely to reflect the limited number of indicators in this construct, and highlights opportunities for further refinement of the Khmer State Mindfulness Questionnaire through the development and evaluation of additional culturally appropriate items. Further, the findings provide largely satisfactory evidence of discriminant validity, while indicating that the distinction between Acceptance and Absence of Aversion may warrant further investigation in future studies. Future research should examine whether additional items can be developed to strengthen the reliability and conceptual coverage of these dimensions. An additional limitation relates to the relatively small number of items loading onto several factors. Three factors in the final model consisted of only two items, which may limit content coverage and reduce the stability of reliability estimates. Two-item factors can be acceptable when supported by strong theoretical and empirical justification. Taken together, the parallel-analysis results, scree plot, and model-fit comparisons supported retention of six factors. Although the six-factor model presented provided the best fit among the models examined, its complexity relative to the number of items and the presence of several two-item factors raise the possibility of over-factoring. Further studies should determine whether the six dimensions replicate or whether a more parsimonious structure provides a more stable representation of the Khmer SMQ.

Lastly, the Khmer SMQ comprises 14 items, with two items removed. Although linguistic and cultural interpretation may have contributed to the performance of these items, our cognitive-interview findings did not identify unresolved comprehension problems following revision. We therefore cannot conclude that translation difficulty was the sole cause. The decision to remove these items was also data-driven to an extent, seeking to ensure all items in the scale were adding to the explanatory power of the final model. As such, future studies should again seek to confirm factor structure for this measure.

Future research should also seek to replicate these findings in broader community and clinical populations, as well as to examine the predictive validity of the Khmer SMQ using longitudinal designs. Cross-cultural comparative studies may also provide further insight into how mindfulness is conceptualised and operationalised across different contexts, contributing to the refinement of both theory and measurement in global mental health.

## Conclusion

This study provides the first culturally adapted Khmer version of the Southampton Mindfulness Questionnaire and offers evidence of its structural validity and internal consistency among Cambodian adults with physical disabilities. The findings suggest that the structure of mindfulness may vary across cultural contexts. The findings highlight the importance of considering cultural meaning systems in the measurement of psychological constructs and support the development of culturally grounded mental health research and interventions in Cambodia.

## Data Availability

The raw data supporting the conclusions of this article will be made available by the authors, without undue reservation.
