# Supplementary material for: Reconceptualising mindfulness in Cambodia: Khmer translation, cultural adaptation, and validation of the Southampton Mindfulness Questionnaire
Source: Front Psychiatry. 2026 Aug 19;17:1901766. doi: 10.3389/fpsyt.2026.1901766 (PMC13533963; doi:10.3389/fpsyt.2026.1901766)
Supplement: Supplementary file 2 [file SupplementaryFile2.docx]

Supplementary File 2

*SMQ Back Translation*

| Original SMQ | Back translation of reconciled Khmer version | Expert panel comments |
| --- | --- | --- |
| Usually when I experience distressing thoughts and images…. |  |  |
| I am able just to notice them without reacting | I just notice it without any reaction |  |
| They take over my mind for quite a while afterwards | It still occurs in my mind for a long time |  |
| I judge the thought/image as good or bad | I decide about that thought is a good thing or bad thing | The word Judge does not have a Khmer equivalent as it would have legal connotations (i.e. a judge in a court of law). ‘Decide” is the best word to reflect the intention of the original text but can be difficult to understand for people with a low level of education. Consensus was reached to use the word “assume” but with the word “decide” in prathenses beside it. |
| I feel calm soon after | I feel calm after a while | Concern that the time period “soon after” was not accurately reflected. When looking at the Khmer version it was agreed that this was not an issue. |
| I am able to accept the experience | I can accept it |  |
| I get angry that this happens to me | I am angry because it happened to me. |  |
| I notice how brief the thoughts and images really are | I notice that thought only occurs in the short period. |  |
| I judge myself as good or bad, depending on what the thought/image is about | I decide that my thought is a good or bad person is depend on that thought. | This could be interpreted as the person themselves being good or bad rather than the thought. The group agreed the Khmer version reflected the original SMQ text and the back translation was not accurate.  The word Judge does not have a Khmer equivalent. As in question 2 we used the word assume” but with the word “decide” in prathenses beside it. (see above) |
| I ‘step back’ and am aware of the thought or image without getting taken over by it | I can notice on that thought through not letting it control my mind. |  |
| I just notice them and let them go | I only notice it and let it go by itself |  |
| I accept myself the same whatever the thought/image is about | I still accept myself no matter what that thought/picture is. |  |
| In my mind I try and push them away | In my mind, I try to force it to go away from me. |  |
| I keep thinking about the thought or image after it’s gone | I keep on thinking of it even it happened for a long time ago. | Concern if the timeframe implied in the original version was accurate. After review of the reconciled Khmer version it was agreed that this was acceptable and no alteration was made |
| I find it so unpleasant I have to distract myself and not notice them | It seems uncomfortable in my mind. I try to think other thing to forget it. | Concern that “unpleasant” and “uncomfortable” have different underlying meanings. Wording in the Khmer version was altered to better reflect an unpleasant thought.  The word think in the backtranslation excludes potential actions that may be taken to distract oneself. The Khmer version was altered to reflect that the person tries to do something else to forget and unpleasant thought rather than thinking of something else. |
| I try just to experience the thoughts or images without judging them | I try to accept it without analyzing it. | Judging something and analysing it reflect different concepts. The Khmer text was changed to read withing making assumptions about it. |
| I lose myself in the thoughts/image | I am so in that thought. |  |
