# Supplementary material for: Reconceptualising mindfulness in Cambodia: Khmer translation, cultural adaptation, and validation of the Southampton Mindfulness Questionnaire
Source: Front Psychiatry. 2026 Aug 19;17:1901766. doi: 10.3389/fpsyt.2026.1901766 (PMC13533963; doi:10.3389/fpsyt.2026.1901766)
Supplement: Supplementary file 3 [file SupplementaryFile3.docx]

Supplementary File 3

SMQ final accepted version in Khmer

|  | **Reconciled translation A&B** | **Final accepted version** |
| --- | --- | --- |
| **Usually when I experience distressing thoughts and images….** | **ជាធម្មតានៅពេលដែលខ្ញុំគិតដល់ការពិបាកចិត្ត/ ទុក្ខព្រួយ…….** | ជាធម្មតានៅពេលដែលខ្ញុំគិតដល់ការពិបាកចិត្ត/ ទុក្ខព្រួយ……. |
| I am able just to notice them without reacting | ខ្ញុំគ្រាន់តែកត់សំគាល់វា ហើយមិនមានប្រតិកម្មតបអ្វីទាំងអស់ | ជាធម្មតានៅពេលដែលខ្ញុំគិតដល់ការពិបាកចិត្ត/ ទុក្ខព្រួយ…….ខ្ញុំគ្រាន់តែកត់សំគាល់វា ហើយមិនមានប្រតិកម្មតបអ្វីទាំងអស់ |
| They take over my mind for quite a while afterwards | វានៅតែបន្តនៅក្នុងចិត្តរបស់ខ្ញុំអស់រយៈពេលក្រោយមក | ជាធម្មតានៅពេលដែលខ្ញុំគិតដល់ការពិបាកចិត្ត/ ទុក្ខព្រួយ…….វានៅតែបន្តនៅក្នុងចិត្តរបស់ខ្ញុំអស់រយៈពេលក្រោយមក |
| I judge the thought/image as good or bad | ខ្ញុំសំរេចថាការគិតនោះជារឿងល្អ ឬរឿងមិនល្អ | ជាធម្មតានៅពេលដែលខ្ញុំគិតដល់ការពិបាកចិត្ត/ ទុក្ខព្រួយ…….ខ្ញុំសន្មត់ (សំរេច) ថាការគិតនោះជារឿងល្អ ឬរឿងមិនល្អ |
| I feel calm soon after | ខ្ញុំក៏មានអារម្មណ៍ស្ងប់ចិត្តវិញ បន្តិចក្រោយមក | ជាធម្មតានៅពេលដែលខ្ញុំគិតដល់ការពិបាកចិត្ត/ ទុក្ខព្រួយ…….ខ្ញុំក៏មានអារម្មណ៍ស្ងប់ចិត្តវិញ បន្តិចក្រោយមក |
| I am able to accept the experience | ខ្ញុំអាចទទួលយកវាបាន | ជាធម្មតានៅពេលដែលខ្ញុំគិតដល់ការពិបាកចិត្ត/ ទុក្ខព្រួយ…….ខ្ញុំអាចទទួលយកវាបាន |
| I get angry that this happens to me | ខ្ញុំខឹងដោយសារវាកើតឡើងលើខ្ញុំ | ជាធម្មតានៅពេលដែលខ្ញុំគិតដល់ការពិបាកចិត្ត/ ទុក្ខព្រួយ…….ខ្ញុំខឹងដោយសារវាកើតឡើងលើខ្ញុំ |
| I notice how brief the thoughts and images really are | ខ្ញុំកត់សំគាល់ថាការគិតនោះដូចជាលេចឡើងក្នុងរយះពេលខ្លីតែប៉ុណ្ណោះ | ជាធម្មតានៅពេលដែលខ្ញុំគិតដល់ការពិបាកចិត្ត/ ទុក្ខព្រួយ…….ខ្ញុំកត់សំគាល់ថាការគិតនោះដូចជាលេចឡើងក្នុងរយះពេលខ្លីតែប៉ុណ្ណោះ |
| I judge myself as good or bad, depending on what the thought/image is about | ខ្ញុំសំរេចថាខ្លួនឯងជាមនុស្សល្អ ឬមិនល្អដោយអាស្រ័យទៅលើការគិតនោះ | ជាធម្មតានៅពេលដែលខ្ញុំគិតដល់ការពិបាកចិត្ត/ ទុក្ខព្រួយ…….ខ្ញុំសន្មត់ (សំរេច) ថាខ្លួនឯងជាមនុស្សល្អ ឬមិនល្អដោយអាស្រ័យទៅលើការគិតនោះ |
| I ‘step back’ and am aware of the thought or image without getting taken over by it | ខ្ញុំអាចសំគាល់ទៅលើការគិតនោះ ដោយមិនឱ្យវាគ្រប់គ្រងចិត្តខ្ញុំបានទេ | ជាធម្មតានៅពេលដែលខ្ញុំគិតដល់ការពិបាកចិត្ត/ ទុក្ខព្រួយ…….ខ្ញុំអាចសំគាល់ទៅលើការគិតនោះ ដោយមិនឱ្យវាគ្រប់គ្រងចិត្តខ្ញុំបានទេ |
| I just notice them and let them go | ខ្ញុំគ្រាន់តែកត់សំគាល់វា រួចហើយទុកឱ្យវាកន្លងផុតទៅដោយខ្លួនឯង | ជាធម្មតានៅពេលដែលខ្ញុំគិតដល់ការពិបាកចិត្ត/ ទុក្ខព្រួយ…….ខ្ញុំគ្រាន់តែកត់សំគាល់វា រួចហើយទុកឱ្យវាកន្លងផុតទៅដោយខ្លួនឯង |
| I accept myself the same whatever the thought/image is about | ខ្ញុំនៅតែទទួលយកខ្លួនខ្ញុំដដែល បើទោះបីជាការគិត/រូបភាពនោះជាអ្វីក៏ដោយ | ជាធម្មតានៅពេលដែលខ្ញុំគិតដល់ការពិបាកចិត្ត/ ទុក្ខព្រួយ…….ខ្ញុំនៅតែទទួលយកខ្លួនខ្ញុំដដែល បើទោះបីជាការគិត/រូបភាពនោះជាអ្វីក៏ដោយ |
| In my mind I try and push them away | នៅក្នុងចិត្តរបស់ខ្ញុំ ខ្ញុំព្យាយាមបង្ខំឱ្យវាចេញទៅឆ្ងាយពីខ្ញុំ | ជាធម្មតានៅពេលដែលខ្ញុំគិតដល់ការពិបាកចិត្ត/ ទុក្ខព្រួយ…….នៅក្នុងចិត្តរបស់ខ្ញុំ ខ្ញុំព្យាយាមបង្ខំឱ្យវាចេញទៅឆ្ងាយពីខ្ញុំ |
| I keep thinking about the thought or image after it’s gone | ខ្ញុំនៅតែបន្តគិតពីវា បើទោះបីជាវាបានកន្លងផុតទៅហើយក៏ដោយ | ជាធម្មតានៅពេលដែលខ្ញុំគិតដល់ការពិបាកចិត្ត/ ទុក្ខព្រួយ…….ខ្ញុំនៅតែបន្តគិតពីវា បើទោះបីជាវាបានកន្លងផុតទៅហើយក៏ដោយ |
| I find it so unpleasant I have to distract myself and not notice them | ខ្ញុំហាក់ដូចជាមិនស្រណុកក្នុងចិត្ត ខ្ញុំក៏ព្យាយាមគិតពីរឿងអ្វីមួយផ្សេងទៅវិញដើម្បីបំភ្លេចវា | ជាធម្មតានៅពេលដែលខ្ញុំគិតដល់ការពិបាកចិត្ត/ ទុក្ខព្រួយ…….ខ្ញុំហាក់ដូចជាមិនស្រណុកក្នុងចិត្ត ខ្ញុំក៏ព្យាយាមបង្វែរទៅធ្វើអ្វីមួយផ្សេងទៅវិញដើម្បីបំភ្លេចវា |
| I try just to experience the thoughts or images without judging them | ខ្ញុំព្យាយាមទទួលយកវា ដោយគ្មានការវិភាគទៅលើវាទេ | ជាធម្មតានៅពេលដែលខ្ញុំគិតដល់ការពិបាកចិត្ត/ ទុក្ខព្រួយ…….ខ្ញុំព្យាយាមទទួលយកការគិតនោះ ដោយមិនធ្វើការសន្មត់ទៅលើវាទេ |
| I lose myself in the thoughts/image | ខ្ញុំស្លុងអារម្មណ៍ក្នុងការគិតនោះ | ជាធម្មតានៅពេលដែលខ្ញុំគិតដល់ការពិបាកចិត្ត/ ទុក្ខព្រួយ…….ខ្ញុំស្លុងអារម្មណ៍ក្នុងការគិតនោះ |
