# Supplementary material for: Reconceptualising mindfulness in Cambodia: Khmer translation, cultural adaptation, and validation of the Southampton Mindfulness Questionnaire
Source: Front Psychiatry. 2026 Aug 19;17:1901766. doi: 10.3389/fpsyt.2026.1901766 (PMC13533963; doi:10.3389/fpsyt.2026.1901766)
Supplement: Supplementary file 5 [file SupplementaryFile1.docx]

Supplementary File 1

 *Figure 1 – Summary of translation and linguistic validation process*
